# eHealth Cloud Security Challenges: A Survey

**DOI:** 10.1155/2019/7516035

**Published:** 2019-09-03

**Authors:** Yazan Al-Issa, Mohammad Ashraf Ottom, Ahmed Tamrawi

**Affiliations:** Yarmouk University, Irbid, Jordan

## Abstract

Cloud computing is a promising technology that is expected to transform the healthcare industry. Cloud computing has many benefits like flexibility, cost and energy savings, resource sharing, and fast deployment. In this paper, we study the use of cloud computing in the healthcare industry and different cloud security and privacy challenges. The centralization of data on the cloud raises many security and privacy concerns for individuals and healthcare providers. This centralization of data (1) provides attackers with one-stop honey-pot to steal data and intercept data in-motion and (2) moves data ownership to the cloud service providers; therefore, the individuals and healthcare providers lose control over sensitive data. As a result, security, privacy, efficiency, and scalability concerns are hindering the wide adoption of the cloud technology. In this work, we found that the state-of-the art solutions address only a subset of those concerns. Thus, there is an immediate need for a holistic solution that balances all the contradicting requirements.

## 1. Introduction

Cloud computing is a relatively new technology that will have a great impact on our lives. Using this technology, it is possible to access computing resources and facilities anytime and anywhere. Healthcare industry is continuously evolving, and the future healthcare model is anticipated to be information-centric. The industry can benefit from the cloud technology to manage change and complexity. This promising technology can help facilitate communication, collaboration, and coordination among different healthcare providers. The cloud can help the healthcare industry deliver more value for the dollar. It can offer fast, flexible, scalable, and cost-effective infrastructure and applications. The cloud can help store, manage, protect, share, and archive electronic health records (EHRs), laboratory information system, pharmacy information system, and medical images. Overall, patients will obtain better care because of up-to-date health records and continuous interactions between different healthcare providers. Beside the lack of standards, regulations, and interoperability problems, the main obstacles that are hindering the wide-scale adoption of the cloud by healthcare providers are the security, confidentiality, and trust issues [1].

Computer security is a growing field in computer science that focuses on protecting computer systems and electronic data against unauthorized access, hardware theft, data manipulation, and against common threats and exposures such as backdoors, denial-of-service (DoS) attacks, and phishing. The objective of applying computer security measures is to attain protection of valuable data and system resources; securing system resources includes protection of a computer system hardware and software, whereas data security is more concerned with protecting data that are stored or transmitted between computer systems, as well as cloud systems. Privacy on the other hand is considered as one of the main objectives of security; it enforces certain rules and principles that regulate to what extent data about individuals or groups can be accessed, gathered, or transmitted to a second or third party. Data ownership is more related to data privacy rather than data security. Privacy could be claimed as a moral right for individuals and groups when using information systems, whereas computer security is not a moral right in itself. Differentiating between computer security and privacy could be more complex, and there are certainly areas of overlap between them [2, 3]. For example, when healthcare providers use secure systems to communicate with patients about their health, rather than transmitting health data via personal e-mail accounts, this type of data communication is an example of a secure implementation. On the other hand, privacy will only attempt to limit the access to patient health records to authorized hospital staff members.

Cloud computing offers opportunities and challenges. Just like every other IT application, the cloud has various security issues and concerns. Since it usually operates in an open and shared environment, it is vulnerable for data loss, theft, and malicious attacks. Weak cloud security is one of the important problems that are hindering the full diffusion of the cloud in healthcare industry. Healthcare professionals have many reasons not to trust the cloud, for example, they cannot give away control over their medical records. Cloud providers usually store their data in different data centers located in different geographic locations. This represents a clear advantage, since data storage on the cloud will be redundant, and in case of force majeure, different data centers will help recover from disasters. On the other hand, this same advantage can pose a security challenge because data stored in different locations will be more prone to theft and loss. In general, there are many security risks associated with the use of the cloud like failure to separate virtual users, identity theft, privilege abuse, and poor encryption are among the security concerns [4].

The goal of this paper is to survey literature and review the state of the art to understand various cloud security challenges and available solutions. This paper tries to answer the following research questions:

- RQ1. What are the cloud computing schemes used in healthcare systems?
- RQ2. What are the security challenges hindering the wide-scale adoption of cloud computing by healthcare providers?
- RQ3. What are the state-of-the-art cloud computing solutions used by current healthcare providers and the security risks associated with those solutions?

The remainder of the paper is organized as follows: Section 2 presents background information about cloud computing.  Section 3 discusses the security requirements needed by healthcare providers for adopting cloud computing.  In Section 4, we survey recent work addressing security risks for eHealth systems using cloud computing. Available security solutions are discussed in Section 5. Finally, our findings and conclusions are summarized in Section 6.

## 2. Cloud Computing

### 2.1. Cloud Definition

There are multiple cloud definitions, different people, different research groups, and different papers that tend to define the cloud in different ways. Nowadays, cloud computing is more of a buzzword rather than a scientific term. According to the National Institute of Standards and Technology (NIST) special publication [5] “*cloud computing is a model for enabling ubiquitous, convenient, on-demand network access to a shared pool of configurable computing resources (e.g., networks, servers, storage, applications, and services) that can be rapidly provisioned and released with minimal management effort or service provider interaction*.” Anyone who delivers technology over the Internet seems to think that he/she is using the cloud technology. Only few papers that use the cloud term exactly meet the NIST models and characteristics.

### 2.2. Cloud Computing Characteristics

According to the official definition, cloud computing has five main characteristics: resource pooling, broad network access, rapid elasticity, on-demand self-service, and measured service [5].

- Shared resources: clients can share resources like networks, servers, storage, software, memory, and processing simultaneously. Providers can dynamically allocate resources according to the fluctuations in demand, and the client is completely unaware of the physical locations of these services.
- Broad network access: the cloud allows a broad access to the network using the Internet from any device.
- Elasticity: the cloud is flexible and configurable. Clients feel that resources are unlimited.
- On-demand self-service: if needed, any customer can automatically configure the cloud without the interference of service technicians. Customers perform scheduling and decides the required storage and computing power.
- Measured service: different cloud services can be measured using different metrics. Detailed usage reports are generated to preserve the rights of customers and providers.

### 2.3. Service Models

Cloud computing has four different service models:

- Software as a service (SaaS): it is the most popular cloud service, and the software resides on the provider platform. The consumer can access the software using a web browser or an application programming interface (API). It follows a pay-per-use business model. Consumers do not need to worry about the software upgrades and maintenance; some limited application configuration capability might be available to consumers. Salesforce and Office 365 are popular examples [5–10].
- Platform as a service (PaaS): it provides development and testing environments. The consumer develops his/her own application on a virtual server and has some control over the application hosting environment, particularly the application and data, making it faster to develop, test, and deploy applications. Cloud Foundry is a good example [11].
- Infrastructure as a service (IaaS): it provides the infrastructure, operating systems, and applications. It is the service of choice for companies that do not have the necessary capital to buy hardware. Customers pay according to consumption.
- Infrastructure is scalable depending on processing and storage needs. The consumer has control over applications, data, middleware, and operating systems but not over the underlying cloud infrastructure. Amazon EC2 is a good example [12].
- Anything as a service (XaaS): it offers a variety of services ranging from personal services to large resources over the Internet [13, 14].

### 2.4. Delivery Models

**Figure 1:**
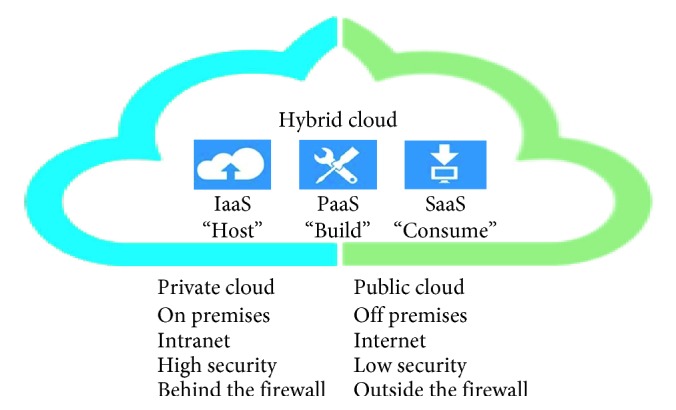
Relationship between delivery and service models.

Cloud computing has five different delivery models:

- Private cloud: it is located on premises, over the intranet, behind the firewall, and usually managed by the same organization that uses it. Their services are offered to the organization employees. Security issues are limited; a good example is VMware [15].
- Public cloud: it is located off premises, over the Internet, and usually managed by a cloud service provider. Their services are offered to the public. It is less secure than the private cloud, some popular public clouds are Dropbox [16], Amazon EC2 [12], and Microsoft Azure [17].
- Hybrid cloud: it combines private and public clouds, and it has trust and confidentiality issues because of the public part. A good example is Rackspace [18].
- Community cloud: it is a group of entities with a common goal, share the cloud; universities usually share a single cloud. A good example is NYSE Capital Markets Community Platform (Figure 1) [19].

### 2.5. eHealth Cloud Benefits

The cloud has many benefits.

- Improved patient care because of the continuous interaction by the patient with different healthcare stakeholders. Patient data are available anytime and anywhere for doctors to analyze and diagnose.
- Cost savings: there is no need to buy expensive hardware and software. Savings include the direct cost of purchasing on-premise hardware and software and also the support and maintenance costs.
- Energy savings: the energy bill will be cut because there is no need for data centers on premises; as a result there, is no need for expensive cooling.
- Robust disaster recovery: in case of emergency, almost all cloud service providers offer a redundant system and services.
- Research: the cloud is a central data repository that can be used to support national medical research, disease control, and epidemics monitoring.
- Solving the scarcity of resources: doctors in remote areas can use telemedicine to perform consultations.
- Rapid deployment: software and hardware systems can be used almost immediately.
- Data availability: data are available for all healthcare stakeholders like physicians, clinics, hospitals, and insurance companies [20, 21].

### 2.6. eHealth Cloud Limitations

The cloud has many limitations:

- Availability and reliability: the service can be slow, interrupted, or down, depending on the strength of the Internet connection. This will largely affect user experiences [6].
- Interoperability: there is a need for standards to achieve proper communication, coordination, and collaboration between different healthcare providers' platforms [7].
- Security and privacy: open and shared environment is prone to data loss and theft [20].
- Legislation and regulations: the wide adoption of cloud computing requires laws, regulations, and ethical and legal frameworks [21].
- Limited control and flexibility: it has limited control over data ownership because of centralization. The cloud applications are often generic, and custom software might be hard to rent [21].
- Vulnerability to attacks: the cloud is prone to different kinds of security attacks [21].

## 3. Common eHealth Security Issues

Nowadays, healthcare is centered on accessing medical records anytime and anywhere. The use of cloud computing paradigm in healthcare facilitates sharing and integration of medical records. However, the cloud computing paradigm offers several benefits; it also poses privacy and security threats to the health data [21]. Essentially, the cloud service providers should deal with security concerns in the cloud to enhance the trust level between the patients and healthcare providers [22–24]. In this section, we discuss important security requirements for eHealth systems to address the arising security and privacy issues hindering the wide-scale adoption of cloud computing by healthcare providers.

There is a long line of research pertaining to the security requirements of healthcare cloud applications. For example, the ISO/TS 18308 standard [25] defines the security and privacy issues for EHRs. The International Medical Informatics Association (IMIA) investigated the issues of data protection and security in healthcare networked systems [26]. The U.S. Department of Health and Human Services (HHS) published a report [26] about personal health records (PHRs), aiming at developing PHRs and PHR systems to put forward a vision that “*would create a PHR that patients, doctors, and other healthcare providers could securely access through the Internet, no matter where a patient is seeking medical care*.” In [27], Bakker et al. present a brief overview on cloud computing security in terms of security considerations, models, threats, and precautions. Avancha et al. [28] examine the privacy requirements of mobile computing technologies that have the potential to transform healthcare industry. Through an extensive survey of literature, Avancha et al. propose a conceptual privacy framework for healthcare applications. In [29], Ardagna et al. present an extensive survey on the interface between cloud security and cloud security assurance. They first provide an overview of the state of the art on cloud security. Then, they introduce the notion of cloud security assurance and analyze its growing impact on cloud security approaches. Finally, they present some recommendations for the development of next-generation cloud security and assurance solutions. Ibrahim et al. [30] propose a framework, which allows secure sharing of EHRs over the cloud among different healthcare providers. The framework ensures the confidentiality, integrity, authenticity, availability, and auditability of EHRs. Along the line, Abbas and Khan [24] present an extensive survey that aims to encompass the state-of-the-art privacy-preserving approaches employed in eHealth clouds. They also classify the privacy-preserving approaches into cryptographic and noncryptographic approaches. Furthermore, the strengths and weaknesses of the presented approaches are reported, and some open issues are highlighted [31] reports on the results of a systematic literature review concerning the security and privacy of EHR systems.

The eHealth system security and privacy concerns do not only deal with abiding by the confidentiality, integrity, and availability (CIA) security model [32]. In [33], Metri and Sarote argue that security threats to the cloud data include spoofing identity via an attacker pretending to be a valid user, tampering with the data that involve malicious alterations and modification of the content, repudiation with the users who deny their signature authenticity after performing an activity with the data, and information disclosure via the exposure of information to unauthorized users [33]. For example, the use and disclosure of the Protected Health Information in the USA should be in accordance with the requirements of the Health Insurance Portability and Accountability Act (HIPAA). The Act considers the confidentiality of health data to be an obligation, not an option [34]. To improve the trust in this relatively new technology, cloud computing applications have multiple security requirements to be fulfilled. Below, we outline the important security and privacy requirements for healthcare application clouds.

### 3.1. Confidentiality

Confidentiality is the act of ensuring that patients health data are kept completely undisclosed to unauthorized entities. Delegating data control to the cloud leads to an increase in the risk of data compromises, as the data become accessible to an augmented number of parties. Due to the increased number of parties, devices, and applications involved, there is an increase in data compromise threats. To make the patient/doctor relationship work effectively, it is necessary for the patient to trust the healthcare system to protect the confidentiality of his/her data. If the patient feels that the information he/she gives to his/her doctor is not protected, and that his/her privacy is threatened, he/she can be more selective about the information he/she will provide to his/her doctor in the future. The threat of data compromise can harm the patient/doctor relationship and hamper the proper medical diagnosis and treatment [35]. For example, an employer may refuse a job if the patient's medical data are disclosed. Confidentiality can be achieved by access control and using encryption techniques.

### 3.2. Integrity

Integrity ensures the health data captured by a system or provided to any entity are accurate and consistent with the intended information and have not been modified in any way [36]. Using the cloud for an important application like eHealth cloud requires assurances of good reliability for the provided services. All eHealth cloud services and data must be error-free. Improper treatment based on erroneous data can have serious consequences on patients' health. The HIPAA Security Rule (*Section 164.312(c) (1) Integrity*) [37] states that covered entities must “*implement policies and procedures to protect electronic personal healthcare information from improper alteration or destruction.*” In a healthcare setting, services that store and manipulate patient data must implement integrity and verification functionality, like nonmedical applications, via the means of a checksum or a hash, before using the data. If the integrity check fails, the healthcare application must report an error and terminate without processing the data. For example, Blough et al. [38] have proposed the use of 21 trees to store public healthcare records.

### 3.3. Availability

For any healthcare cloud system to serve its purpose, the information must be available all the time. An important and often overlooked aspect in the eHealth system is the availability of data in critical situations, including the ability to carry on operations even when some authorities misbehave and the ability to continue operations even in the possibility of a security breach. High-availability systems should prevent service disruptions due to power outages, hardware failures, system upgrade, and denial-of-service attacks. It should also be able to preserve the usability of healthcare records after enforcing HIPAA security and privacy rules.

### 3.4. Ownership and Privacy of Healthcare Information

In general, the owner is defined as the creator of the information. Establishing information ownership is necessary for protection against unauthorized access or misuse of patient's medical information. Ownership of healthcare information can be protected through a combination of encryption and watermarking techniques that result in secured healthcare information that cannot be transmitted, accessed, or released without the mutual acceptance of all entities involved in the ownership/creation of the healthcare information. Patients can allow or deny the sharing of their information with other healthcare practitioners [33]. To implement patient data sharing in a healthcare system, patient may grant rights to users based on a role or attributes held by the respective user to share specific healthcare data with that user.

### 3.5. Authenticity

Authenticity in general refers to the truthfulness of origins, attributions, commitments, and intentions. It ensures that the entity requesting access is authentic. In healthcare systems, the information provided by the healthcare providers and the identities of the entities using such information must be verified via the Authentication Act [39]. The authentication of information can pose special problems, like man-in-the-middle attacks, and is often mitigated with a combination of usernames and passwords. Most cryptographic protocols include some form of endpoint authentication specifically to prevent man-in-the-middle attacks. In a healthcare system, both healthcare information offered by providers and identities of consumers should be verified at every access.

### 3.6. Nonrepudiation

Repudiation threats are concerned with the users who deny their signature authenticity after accessing health data [40]. For instance, in the healthcare scenario, neither the patients nor the doctors can deny their signature authenticity after misappropriating the health data. Just like electronic commerce, healthcare cloud applications can leverage digital signatures and encryption to establish authenticity and nonrepudiation.

### 3.7. Audit

Auditing is a security measure that ensures the safety of a healthcare system. Audit means recording user activities of the healthcare system in chronological order, such as maintaining a log of every access and modification of data.

Both HIPAA and Health Information Technology for Economic and Clinical Health (HITECH) require users within the healthcare provider's organization to be held accountable for their actions when handling patients' protected health information. There are different approaches to maintaining audit controls for such information; e.g., Integrating the Healthcare Enterprise (IHE) specifies a profile for the Audit Trail that contains sufficient information to answer questions such as: “*For some user: which patient's records was accessed? For some patient's record: which users accessed it? What user authentication failures were reported?”* Such approaches could help administrators mitigate insider threats by ensuring detection of unauthorized access and illegal disclosure of healthcare records. Auditing could also help detect attempts by hackers to break into a public healthcare cloud system and help administrators detect potential vulnerabilities in the system [41].

### 3.8. Access Control

Access control is a mechanism for controlling access to a patient's public health information that restricts access to legitimate entities only. The access control policy is typically based on the privilege and right of each authorized practitioner by patient or a trusted third party. Several solutions have been proposed to address the security and access control concerns. Role-based access control (RBAC) and attribute-based access control (ABAC) are the most popular models for healthcare application clouds [42–45].

### 3.9. Data Remanence and Freshness

Data remanence refers to the residual representation of data that have been in some way nominally erased or removed. Data remanence may cause an unintentional data confidentiality attack. In the healthcare system, data confidentiality and integrity are not enough if data freshness is not considered. Data freshness implies that the patient health records must be fresh and up-to-date. Delays in storage and sending outdated notifications result in data inconsistency, especially in critical situations.

### 3.10. Anonymity

Anonymity refers to the state where a patient cannot be identified from his/her public health records acquired for research and quality improvement. For instance, identities of the patients can be made anonymous when they store their health data on the cloud so that the cloud servers could not learn about the identity. The HIPAA Privacy Rule states that covered entities may use or disclose public healthcare information that is deidentified without restriction [46, 47]. Covered entities that seek to release such data must determine that the information has been deidentified using either statistical methods to verify deidentification or by removing certain parts of the data. Under the HIPAA Privacy Rule, a covered entity can deidentify public healthcare record by removing all 18 elements that could be used to identify the patient or the patient's relatives, employers, or household members. The rule also requires the covered entity to have no actual knowledge that the remaining information could be used alone or in combination with other information to identify the patient. Some of the 18 identifiable elements are the patient's name, geographical information such as ZIP code, phone number, all elements of dates except the year, and biometrics. Anonymization in healthcare data setting is an active area of research, with extensive literature; Appari et al. provide a useful overview by citing several research efforts aimed at anonymizing patient data: global and local recoding, confidential audits of medical record access, microaggregation, and data perturbation [48–52].

### 3.11. Unlinkability

Unlinkability refers to the use of resources or items of interest multiple times by a user without other users or subjects being able to interlink the usage of these resources. This means that the probability of those items being related from the attacker's perspective stays the same before and after the attacker's observation [53].

### 3.12. Cloud Multitenancy

Clouds were built for several reasons of which some of the most important reasons were shared computing, shared memory, and shared storage. Cloud providers deploy multitenancy as a standard to achieve efficient utilization of resources, while decreasing cost. Thus, security threats prevail to data access and management to secure data sharing and integration. To deliver secure multitenancy, there should be isolation among patients' data [54, 55].

### 3.13. Secure Transmission

The HIPAA Security Rule (Section 164.312(e) Transmission Security) states that covered entities must “implement technical security measures to guard against unauthorized access to electronic protected health information … transmitted over an electronic communications network” [37]. The 2009 HITECH Act extends this rule to business associates. Although HIPAA's rule covers communication between HIPAA-covered entities, the concern here is an adversary who wishes to obtain confidential medical information from observing the network communications between two communicating nodes. For example, the adversary may inspect the network packets and obtain sensitive medical data; this problem can be solved by encrypting all communications. For example, most emerging services use Hypertext Transfer Protocol (HTTP) over Secure Sockets Layer (SSL). Even if the network traffic is encrypted, in some settings, it is possible for a clever adversary to use traffic analysis via the study of the size and timing of network packets to determine characteristics of the traffic. This is called side-channel attacks. Many solutions require the addition of delays (to defeat timing analysis) or padding (to defeat packet-size analysis) [46, 47, 56–58]. Consequently, these ad hoc solutions pose non-negligible overhead on system performance and resource usage.

## 4. Recent Work in eHealth Security

There is a vast amount of work that has been done with regard to addressing security and privacy risks in eHealth. In this section, we survey recent work and proposed secure eHealth system architecture.

Hamid et al. target the confidentiality of healthcare patient's multimedia data in the cloud by proposing a tri-party one-round authenticated key agreement protocol based on bilinear pairing cryptography. The proposed protocol can generate a session key among the participants to communicate securely. Finally, the private healthcare data are accessed and stored securely by implementing a decoy technique with a fog computing facility. Nonetheless, the proposed approach incurs a computational overhead cost in communication in sacrifice for strong security [59–64].

Marwan et al. propose a novel method based on Shamir's Secret Share Scheme (SSS) and multicloud concept to enhance the reliability of cloud storage in order to meet security requirements to avoid loss of data, unauthorized access, and privacy disclosure. The proposed technique divides the secret data into many small shares so that one does not reveal any information about medical records. Besides mutlicloud architecture, data are spread across different cloud storage systems. In such a scenario, cloud consumers encrypt their data using SSS technique to ensure confidentiality and privacy. Therefore, the healthcare data are split into various shares, so that data confidentiality is guaranteed. On contrary, the article does not discuss any aspects of the optimal number of shares for the incurred trade-off between efficiency and security. It does not discuss the quality analysis of recovered healthcare data [65, 66].

Galletta et al. present a system developed at Instituto di Ricovero e Cura a Carattere Scientifico (IRCCS) that is claimed to address the patient's data security and privacy. The presented system is based on two software components, the anonymizer and splitter. The first collects anonymized clinical data, whereas the second obfuscates and stores data in multiple cloud storage providers. Thus, only authorized clinical operators can access data over the cloud. They present a case study that uses magnetic resonance imaging (MRI) data to assess the performance of the implemented system [67]. Alexander et al. propose a privacy-aware system and anonymization techniques for data publishing on cloud for PHRs. The proposed system uses *k*-anonymity and Advanced Encryption Standard (AES) [68–70].

Smithamol et al. address the data confidentiality and access privacy by proposing a novel architecture for the outsourced health records. The proposed model uses partially ordered set for constructing the group-based access structure and Ciphertext-Policy Attribute-Based Encryption (CP-ABE) to provide fine-grained medical records access control. The proposed approach minimizes the computational overhead and the overall encryption time. Nevertheless, the performance analysis shows the efficiency of the proposed model, making it suitable for practical use [71, 72]. Sneha and Asha propose to use *k*-anonymity for privacy preserving on eHealth records [73].

Ibrahim et al. provide a comprehensive solution to secure access to privacy-sensitive EHR data through (1) a cryptographic role-based technique to distribute session keys using Kerberos protocol, (2) location- and biometrics-based authentication method to authorize the users, and (3) a wavelet-based steganographic technique to embed EHR data securely in a trusted cloud storage. The article also shows the resilience of the proposed solution to man-in-the-middle and replay attacks. However, they did not analyze the scalability of the approach and its resilience to other significant security risks including integrity and availability of the data as well as the computational overhead [74].

Shah and Prasad list various methods of encryption and also addresses security and privacy challenges in healthcare cloud by deploying a novel framework with cloud-based privacy-aware role-based access control (CPRBAC) model. The side goal is to reduce computational complexity and communication overhead. However, there is no qualitative analysis discussion on the efficiency of the approach and its mitigation to security and privacy attacks [75].

Supriya and Padaki survey several healthcare security lapses pertaining to nonrepudiation, CIA model, and what it means to stakeholders in the healthcare industry. They also discuss few proven operational strategies and risk management methodologies and discern what the industry can do to mitigate such security risks and privacy threats. The article classifies the security threats posed on healthcare clouds into three high-level categories, including network, system care and protection, and compliance with standard acts and rules [76].

The article discusses important concepts related to EHRs sharing and integration in healthcare clouds and analyzes the arising security and privacy issues in access and management of EHRs. The article presents several basic security and privacy requirements for application clouds: ownership; authenticity; nonrepudiation; patient consent and authorization; integrity and confidentiality; and availability, archiving, and auditing. Then they present an EHR security reference model for managing security issues in healthcare clouds, which highlights three important core components in securing an EHR cloud: secure collection and integration, secure storage and access management, and secure usage model. Finally, they illustrate the development of the proposed EHR security reference model through a use-case scenario and describe the corresponding security countermeasures and possible security techniques [77].

Ibrahim et al. propose a framework, which allows secure sharing of EHRs over the cloud among different healthcare providers. In the proposed framework, public key infrastructure (PKI) is used to maintain authentication between participating healthcare providers and the EHR sharing cloud. The proposed framework claims that it ensures the confidentiality, integrity, authenticity, availability, and auditability. It also claims that it meets the security standards defined in the technical safeguards of the HIPAA Security Rule [30].

Löhr et al. present security architecture for establishing privacy domains in eHealth infrastructures. This architecture is based on Trusted Virtual Domains (TVDs) that extend the protection of privacy-sensitive data from centrally managed secure networks to the client platforms of the end-users. However, there are still open research challenges not addressed by the presented architecture, including anonymity, nonrepudiation, and inability of the patient to authenticate [78, 79].

## 5. Available eHealth Security Solutions

Security and privacy issues are among the most talked about topics in information technology and communications fields. Many healthcare providers use cloud technology with caution due to the risks involved such as unauthorized use or access to private and sensitive health data. To mitigate security and privacy concerns, some guidelines and recommendations must be addressed by cloud service providers. All solutions suggested in literature are not holistic in nature; they partially address some of the cloud security problems discussed in Section 3. In the following subsections, we discuss the available solutions from regulatory and technical aspects.

### 5.1. Regulatory Aspects

Standards are usually created to describe accepted characteristics of a product or service by experts from organizations and scientific institutions. These standards are documented and published to represent a consensus on characteristics such as quality, security, and reliability that should remain applicable for an extended period of time. The standards goal is to support individuals and companies when procuring goods and services. Cloud service providers can boost their reputation by complying with standards. Different countries developed multiple standards to guarantee cloud privacy and security. Below we review US (e.g., HIPAA and HITECH) and international standards (e.g., ISO/IEC 27000 and General Data Protection Regulation (GDPR)).

### 5.2. US Standards

#### 5.2.1. HIPAA

HIPAA is a legal framework for securing healthcare systems. HIPAA required the Secretary of the HHS to set rules, guidelines, and acts to protect the privacy and security of health data. As a result, HHS issued HIPAA Security Rule and HIPAA Privacy Rule. The main goal of the Security Rule is to protect the individual's health data in balance with permitting technology bodies to adopt information technology advancement to benefit healthcare services and produce quality services for individuals and healthcare providers. Specifically, The Security Rule requires technology bodies to use administrative, technical, and physical safeguards to protect health data by ensuring the confidentiality, integrity, and availability health data; protect health data against all threats to the security or integrity of data; provide protection against unauthorized use of health data; and ensure technology bodies and service providers compliance. The HIPAA Privacy Rule aims to set standards and guidelines to protect patients' medical records. The rule implements appropriate safeguards to protect the privacy of PHR, provide limitation on data uses without patient authorization, grant patients the rights to examine and obtain a copy of their medical records, and allow patients to amend incorrect information [80, 81]. Other approaches used to enhance the security level and confidentiality are as follows. First, individual identification is deleted during data collection (anonymous data). Second, individual identification is initially recorded during data collection and eventually removed. In this type of identification, there is a chance to reidentify the patient because patient information has been recorded at some stage (anonymized data). However, the removal of personal health data requires the removal of data elements like medical record numbers, social security numbers, Internet Protocol (IP) addresses, health plan beneficiary numbers, e-mail addresses, web Universal Resource Locators (URLs), fax numbers, account numbers, and device identifiers. Removing these data to meet De-identification Act can affect the outcome of data analysis. Third, encoding and encrypting data; however, there is a chance to reveal the encryption key using advanced computer technology. Privacy advocates and data regulators are gradually complaining about data collection and data usage in the Big Data era, and they call for a sophisticated protocol that balance between individual privacy and research benefits [82–86].

#### 5.2.2. HITECH

The HITECH Act is a healthcare legislation created by the HHS meant to widen and accelerate the adoption of EHR and to improve the performance of healthcare systems. The HITECH Act motivates and rewards healthcare providers by offering incentives and grant programs and increases public trust in EHR by setting appropriate privacy and security measures. It also encourages investments in developing healthcare systems. HITECH Act regulations were motivated by the lack of financial resources, shortage in technical expertise, and the lack of a secure infrastructure for exchanging healthcare information [87]. For example, to overcome the financial obstacles, healthcare providers may obtain up to $63,750 in extra payments if they become an effective user of EHR between 2011 and 2021. In addition, the US government has reserved about $650 million under the HITECH Act to create a national infrastructure for health information exchange called the Nationwide Health Information Network, and they will establish a set of standards and policies that will ensure secure exchange of health information on the web [87, 88].

### 5.3. International Standards

#### 5.3.1. ISO/IEC 27000-Series

The ISO/IEC 27000-series [89] is a series of standards reserved for addressing information security concerns. The series is jointly published by the International Organization for Standardization (ISO) and the International Electrotechnical Commission (IEC). The ISO/IEC 27000-series brings best practices on information security management within an Information Security Management System (ISMS) [90, 91].

**Figure 2:**
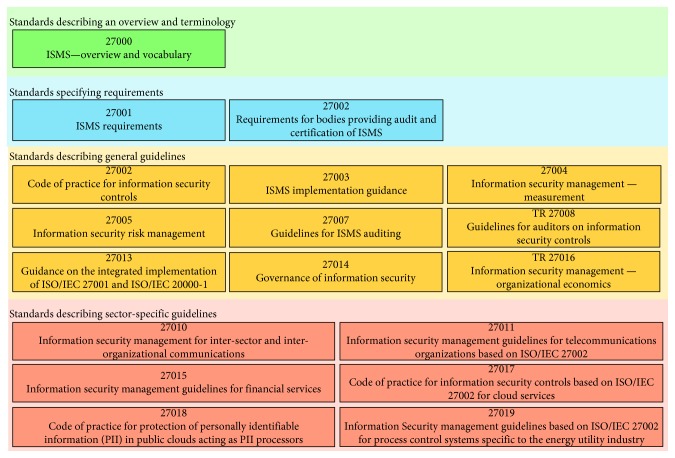
ISO/IEC 27000-series standards categories.

Figure 2 shows the relationship between different ISO/IEC 27000-series standards. It shows that the ISO/IEC 27000-series standards can be grouped into 4 different categories based on the purpose and scope of each standard. The categories are (1) *vocabulary and terminology* category that describes the fundamentals of ISMS and defines related terms, (2) *requirement standards* category consists of the standards that provide requirements and guidelines for the development and operation of an ISMS, (3) *guideline standards* category provides a practical implementation guidance for securing information from different angles, (4) *sector-specific guideline standards* category consists of standards that appeal to different industry sectors such as telecommunication, finance, etc. Below, we present the details of ISO/IEC 27001 and ISO/IEC 27002. The other standards in the ISO/IEC 27000-series are used to guide and support the ISO/IEC 27001/27002 auditing and certification process.

The ISO/IEC 27001 specifies the requirements for establishing, implementing, operating, monitoring, reviewing, maintaining, and improving formalized ISMS and their alignment with the organization's strategic goals. ISO/IEC 27001 certification secures information assets and restores patients trust in cloud service providers. The standard adopts the Plan-Do-Check-Act (PDCA) model to structure all ISMS processes. The model ensures that ISMS is established, implemented, assessed, measured where applicable, and continually improved. Currently, the standard defines 114 controls grouped into 14 control objectives. Control objectives include communications security, cryptography, and information security incident management. Overall, accredited registrars are reporting an increased demand on ISO/IEC 27001 certification from service providers [92].

**Figure 3:**
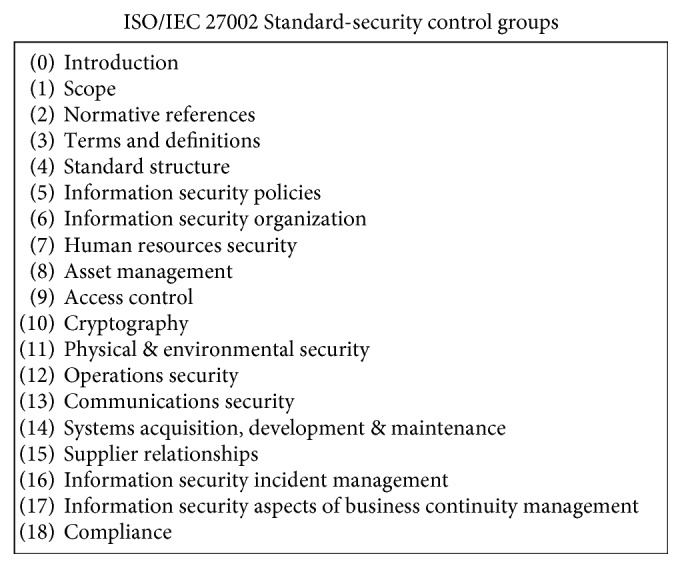
ISO-27002 best practice topics [94].

The ISO/IEC 27002 [93] standard concentrates on security during system planning and development stages. The standard is structured logically around groups of related security controls.  Figure 3 summarizes 19 best practices [94]. For example, section 10 in Figure 3 states “*there should be a policy on the use of encryption, plus cryptographic authentication and integrity controls such as digital signatures and message authentication codes, and cryptographic key management*” [94]. Section 13 in Figure 3 includes controls on network security management and information transfer. It should be noted that ISO/IEC 27002 is a code of practice to adhere to, not a formal certification as ISO/IEC 27001 [95].

#### 5.3.2. EU General Data Protection Regulation (GDPR)

GDPR is the European Union (EU) primary tool that regulates the protection of EU citizens individual data. The recent rule enhances the privacy rights of individuals and gives authorities a greater power to act against noncompliant organizations. The new rule protects personal data of 500 million EU citizens in all 28 EU member states. They are meant to harmonize local data privacy laws across Europe. The new rules are going to help fight terrorism; it is also going to gain people's trust in various digital services, giving a strong boost to the economy [96, 97].

GDPR started recently on 25/5/2018 and replaced the old data protection regulation; it gives consumers more control over their data, it protects the free movement of personal data within the European Union, and it also regulates the export of personal data outside the EU. This act is applicable worldwide, and it applies on every organization that is handling EU citizens' data. It applies on EU organizations like data controllers and data processors that collect or process the personal data of EU residents; it also applies on data controllers and data processors that reside outside the EU if they offer goods and services to data subjects that reside in the EU [96, 98, 99].

Cloud service providers should demonstrate compliance by maintaining a log of all data processing activities. They should apply the appropriate personal and organizational measures. Unlike the old Data Protection Directive, noncompliant organizations will face severe punishment for data breaches; the most serious infringement can cost a company twenty million Euros or up to 4% of the annual worldwide turnover, whichever is greater [96]. The law opens the door for compensation claims for suffered damages, including reputational damages [99]. Under the new regulations, companies should ask for explicit consent from consumers, customers also have the right to opt out, and businesses should keep a log of all consumer consents [100]. Privacy by design means that service providers should design their processes to accommodate consumer privacy, should comply with protection laws, and should monitor what personal data they hold, where it came from, whom they share it with, and where do they store a customer data. In addition, data must be used for the reason it was collected. The rights of data subjects are expanded in the new regulation. The new regulation gives consumers the right to be forgotten, data must be permanently erased if requested. Breach notification is mandatory in all member states, the new act expects a company to report data breaches to the regulator and customer within 72 hours or face severe penalties. Organizations should also have plans in place to recover from security breaches when they occur. Consumers have the right for their data to be available in a portable “commonly use and machine readable” format. They also have the right to transfer their data to a different provider [96, 98, 101, 102].

### 5.4. Technical Aspect

#### 5.4.1. Patient-Centric Approach

**Figure 4:**
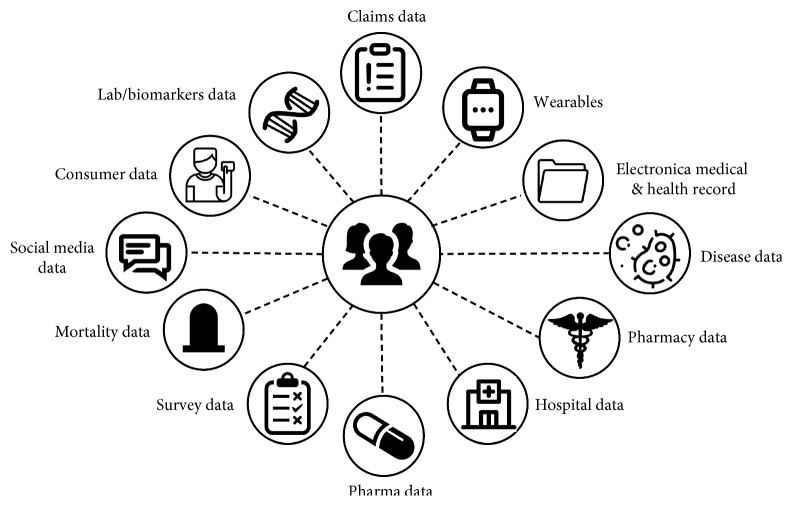
Information-centric healthcare model [107].

It is a community of healthcare systems where patients can store, access, update, and share their health data [103]. Patient-centric offers secure storage and administration of patients EHRs, which could be utilized for disease treatment, research, and other applications. Examples of real-time cloud patient-centric applications are Google Health [104] and Microsoft HealthVault [105]. Both applications implement a centralized architecture where patients store and update health data in EHR system, and patients have full control over their data [77]. Since patient PHR stored in the cloud or at third party, there have been wide privacy issues because patient private health data could be used by third-party servers or unauthorized users. To assure the patients' privacy and to enhance security, it is highly recommended to encrypt patient data before outsourcing [106]. Encrypting data takes time and may affect performance (Figure 4).

The task of aggregating health records from different sources in a single repository is a complex task since the aggregator needs to use different standards and protocols to guarantee interoperability between different stakeholders. On the other hand, the use of different standards makes it hard to secure the application and makes it prone to security breaches. The problem with the patient-centric approach to solving the security problem is the contradicting requirements. Giving the power to the patients to decide who can access their records might prevent a doctor from accessing those records in case of an emergency. Applying multilayer security measures to guarantee that only authorized users can access the system might slow the system down and collides with the doctors need for fast and quick systems. Added security measures will negatively affect the user experience. The fundamental need for different parties to access the patient data makes the patient data more vulnerable to security breaches. Giving the patient the ability to edit his/her own medical records might collide with the doctor's requirement to guarantee data originality [108, 109].

### 5.5. Encryption Techniques

Cloud computing and the widespread connectivity have increased the risk of data breaches. Health data are highly sensitive, and safeguarding these data is a high priority for individuals, healthcare providers, and cloud services providers. Encryption is considered an important part of the security policy of organizations and service providers because it can circumvent intruders gaining value from data. Encryption is a technique that is used to scramble cleartext data into ciphertext with a key. The key is used later by authorized party to decode data to the original form. Encryption uses a computer algorithm to decode data and generate the key where knowing or guessing the key is highly difficult. Ciphertext (encrypted data) is considered more secure from the clear text data, and it prevents unauthorized users from obtaining a value or meaning from accessing the data. In cloud computing, encryption must be considered during data in motion, data at storage, and during data deletion [110].

Encrypting of data in transit is the process of encrypting data at one location, transferring it over the network, then decoding data at the cloud. It became an important process because unauthorized eyes could have access to the data on the way, causing data integrity issue (data could be modified or stolen during transfer). The Transport Layer Security (TLS) has been utilized to secure communication between web applications. TLS reserves an encrypted channel to establish negotiations between senders and receivers to send the cipher, then transfer the key using public key cryptography [111].

In cloud computing, sensitive data-in-rest suffers many threats that can cause data leakage. Self-encrypting drive (SED) is a hard drive that contains internal circuits that encrypts and decrypts all data automatically and uses authentication procedure when the host system is powered on [110]. Encryption key management is crucial for data-in-rest encryption; therefore, it is highly recommended to maintain control of all keys, store keys externally, and maintain transparent encryption to the users [112]. The goal of secure data-deletion encryption is to protect data deletion against expert attackers, so that securely deleted data are not recoverable. If the attacker has a backup version of deleted encrypted data, then the system admin and users must guarantee that the corresponding decoding key is also strictly deleted to prevent attacker from decoding data thereafter [113].

Finally, with the increasing demand for better performance and scalability of eHealth systems and the wide adoption of IoT (Internet of things), emerging technologies such as edge and fog computing are used to complement cloud computing. Edge computing aims at processing data at the edge of the network rather than processing data at the data center as in traditional eHealth cloud solutions. Fog computing aims to process data as close as the service invoker (e.g., IoT wearable health devices), which could help reduce unnecessary latency in eHealth services. Overall, the goal of using edge and fog computing technologies is to (1) enable fast and prompt interactions for responsive healthcare services as the latency imposed in such services could define the margin between death and life in some critical cases and (2) an increase in the computing power for such services without overwhelming the data center. However, the security challenges imposed by these technologies are inventible and alarming compared to traditional eHealth cloud technologies. For example, the fog and edge computing technologies rely on distributed data processing across many locations rather than within a small and limited number of locations in case of cloud storage and traditional data centers. Therefore, the security in edge and fog technologies should be tightened and enhanced by (a) utilizing the state-of-the-art security mechanisms within the edge computing communication environment, (b) encrypting all data (in-move and in-rest), and (c) multifactor authentication access [114–119]. In the future, we are planning to survey the state-of-the-art security mechanisms for eHealth systems on emerging fog and edge technologies and compare those mechanisms with security mechanisms in eHealth cloud systems.

## 6. Conclusion

Security is one of the main problems that hinder the fast adoption of the cloud computing technology in the healthcare industry. The strengths and benefits of cloud computing far exceed its dangers and threats. Security requirements are increasingly difficult to meet without a significant investment in infrastructure and manpower. The dilemma is that security is negatively proportional to consumer convenience. In other words, the more sophisticated the security measures, the less comfortable the consumers, and as a result, they are going to be less inclined to use the cloud service. In this paper, we found that the surveyed solutions are not holistic in nature, those approaches partially solve the security challenge. Most of those solutions address part of the problem, and they failed to balance all contradicting security requirements. The problem is that a gain obtained in one dimension causes a loss in another dimension. In the future, we will propose a holistic solution that attempts to balance all contradicting requirements.

Migration of an organization data to the cloud is a strategic and complex decision. Before moving data into the cloud, the security challenges should be mitigated. A good cloud service provider should monitor the protected health data life cycle. Before selecting a cloud service provider, the following different questions should be asked: Is the provider ISO/IEC 72001 certified? Is the provider compliant with the security and privacy regulatory acts? Is the provider staff trained on risk and crisis management? Whether the provider performs periodic security checks? Is the service provider willing to sign a strong HIPAA Business Associate Agreement (BAA) that contains severe punishment in case of terms violation. Different security measures like firewalls, intrusion detection, and the type of encryption and authentication techniques should be also checked.
